# Ethical Challenges in Promoting the Implementation of Preventive Interventions: Report of the SPR Task Force

**DOI:** 10.1007/s11121-018-0912-7

**Published:** 2018-06-23

**Authors:** Bonnie J. Leadbeater, Tom Dishion, Irwin Sandler, Catherine P. Bradshaw, Kenneth Dodge, Denise Gottfredson, Phillip W. Graham, Sarah Lindstrom Johnson, Mildred M. Maldonado-Molina, Anne M. Mauricio, Emilie Phillips Smith

**Affiliations:** 1 Department of Psychology, University of Victoria, Cornett Building A241, 3800 Finnerty Road (Ring Road), Victoria, BC V8P 5C2 Canada; 2 Department of Psychology, REACH Institute, Arizona State University, 900 S. McAllister Rd, Tempe, AZ 85287-1104 USA; 3 Department of Psychology, Arizona State University, 900 S. McAllister Rd, Tempe, AZ 85287-1104 USA; 4 Curry School of Education, University of Virginia, 112-D Bavaro Hall, 417 Emmet Street South, PO Box 400260, Charlottesville, VA 22904-4260 USA; 5 Sanford School of Public Policy, Duke University, 222 Rubenstein Hall, Durham, NC 27708 USA; 6 Department of Criminology and Criminal Justice, University of Maryland, 2220 LeFrak Hall, College Park, MD 20742 USA; 7 Drugs, Violence, and Delinquency Prevention Program, Center for Justice, Safety, and Resilience, RTI International, 3040 Cornwallis Rd, Raleigh, NC USA; 8 School of Social and Family Dynamics, Arizona State University, PO Box 873701, Tempe, AZ 85287 USA; 9 University of Florida, Institute for Child Health Policy and Family Data Center, College of Medicine, Gainesville, FL 32608 USA; 10 REACH Institute, Department of Psychology, Arizona State University, P.O. Box 876005, Tempe, AZ 85287-6005 USA; 11 Human Development and Family Science, University of Georgia, 305 Sanford Drive, Athens, GA 30602 USA

**Keywords:** Implementation, Dissemination, Evidence based interventions, Ethical challenges, SPR Task Force

## Abstract

Prevention science researchers and practitioners are increasingly engaged in a wide range of activities and roles to promote evidence-based prevention practices in the community. Ethical concerns invariably arise in these activities and roles that may not be explicitly addressed by university or professional guidelines for ethical conduct. In 2015, the Society for Prevention Research (SPR) Board of Directors commissioned Irwin Sandler and Tom Dishion to organize a series of roundtables and establish a task force to identify salient ethical issues encountered by prevention scientists and community-based practitioners as they collaborate to implement evidence-based prevention practices. This article documents the process and findings of the SPR Ethics Task Force and aims to inform continued efforts to articulate ethical practice. Specifically, the SPR membership and task force identified prevention activities that commonly stemmed from implementation and scale-up efforts. This article presents examples that illustrate typical ethical dilemmas. We present principles and concepts that can be used to frame the discussion of ethical concerns that may be encountered in implementation and scale-up efforts. We summarize value statements that stemmed from our discussion. We also conclude that the field of prevention science in general would benefit from standards and guidelines to promote ethical behavior and social justice in the process of implementing evidence-based prevention practices in community settings. It is our hope that this article serves as an educational resource for students, investigators, and Human Subjects Review Board members regarding some of the complexity of issues of fairness, equality, diversity, and personal rights for implementation of preventive interventions.

The field of prevention science has advanced considerably over the past four decades. In 1988, a task force was assembled to identify prevention programs that “work” (Price et al. 1989). Nearly 25 years later, more than 100 evidence-based prevention programs (EBPs) have been established as promising or effective (Hawkins et al. 2015; National Research Council/Institute of Medicine 2009; Sandler et al. 2014; Sloboda et al. 2014). The success of preventive interventions has catapulted prevention scientists into new roles as implementers of EBPs. Implementation activities range from consulting on the selection of an EBP for a given community or agency to more extensive community engagement in the long-term work of implementing EBPs, both of which can affect large-scale changes that aim to advance public health and well-being. New roles also evoke new pressures and ambiguities in the inevitable tensions between scientific rigor and practical need and wisdom (see Weisz et al. 2014). The changing role of the prevention scientist creates new ethical challenges for both researchers and practitioners (Cargill et al. 2016; McCarthy 2016). At this juncture, it is timely to promote the discussion of emerging ethical issues related to scaling up EBPs, especially given the dearth of professional and scientific guidelines.

Current definitions of implementation science have evolved from research aiming to improve knowledge about the effectiveness of implementation strategies. For example, Lavery (2016) proposed that “implementation science, in essence, is about trying to use research strategies to gain a better understanding of the complex array of structural and human factors that can determine whether new programs or interventions will work as intended” (p. 1). Similarly, the National Institutes of Health (as cited in Sloboda et al. 2014) defines implementation science as “the study of methods to promote the systematic uptake of clinical research findings and other evidence-based practices into routine practice and hence to improve the quality (effectiveness, reliability, safety, appropriateness, equity, efficiency) of health care” (p.293).

In such university-based implementation research activities, Institutional Review Boards (IRBs) systematically review research plans for human subject protection around data collection and storage, data linking, analyses, and partnerships. However, implementation activities do not always involve systematic research, and therefore, review processes do not always extend to implementation or scale-up activities. In general, implementation activities in prevention science have the broad goals of promoting awareness, fostering dissemination, adoption, and adaption of EBPs. Prevention scientists may be working to build the capacity of community service providers to utilize and sustain their investments in EBPs, with the goal of fostering change that benefits the public good. Implementation activities of prevention researchers, thus, connect them with user group members, practitioners, and stakeholders who may come from a variety of non-research settings including schools, communities, social and public health agencies, and government or health care organizations. These institutions, in turn, often represent or work with individuals from a variety of social, economic, and ethnic backgrounds, including those who are vulnerable due to young age, poverty, sexual orientation, disabilities, minority, immigrant, or aboriginal status.

Reliance on untested programs can be fraught with concerns and can have negative consequences. However, the evidence base for what intervention works, for what population, under what circumstance, and for whom has many gaps (Gottfredson et al. 2015). Hence, both prevention scientists and the potential users of EBPs may face hard decisions in contexts of considerable uncertainty. With some important exceptions (e.g., Conduct Problems Research Group 2010; Dymnicki 2014), promising interventions are typically supported by small-scale efficacy or effectiveness studies, which have been conducted with circumscribed, typically predominately Caucasian groups. Such samples limit the robustness, generalizability, and certainty of their impact with other groups (Gottfredson et al. 2015). Additionally, potential users of preventive interventions may also be hoping to address complex and widespread societal issues while also experiencing serious constraints imposed by financial, staffing, and resource limitations. Such limitations can hinder the fidelity and quality of implementation of a program and possibly dampen the expected outcomes. When implementation activities are undertaken, conflicts between the priorities of the prevention scientist, practitioners, and/or program users may result in ethical dilemmas (i.e., difficult decisions and hard choices) concerning conflicts of interest, potential risk encountered, inequities in benefits received, social justice, and confidentiality.

In 2015, the Society for Prevention Research (SPR) commissioned Irwin Sandler and Tom Dishion to organize a series of SPR roundtables and to establish a task force to explore and articulate the salient ethical issues that occur as prevention researchers are involved in delivering, adapting, disseminating, and implementing preventive interventions. This article summarizes the activities and findings of the task force and articulates some of the ethical issues that can arise as prevention scientists and practitioners engage with community stakeholders in the implementation of preventive interventions. In considering these activities and ethical challenges, we identify broad ethical principles that can guide ethical behavior and identify questions that could guide deliberation and navigation of challenges.

A growing number of publications include insightful discussion of ethics related to specialized areas of prevention science research including interventions involving genetic testing (Clayton 2003; Fisher & Harrington McCarthy 2013) and the use of internet technology and social media (Conway 2014; Pisani et al. 2016; Mikal et al. 2016). Building on prior work and existing frameworks from other fields, we identify distinctive implementation activities of prevention scientists and practitioners, ethical dilemmas that can arise in the course of implementation and scale-up activities, and suggest ethical principles and questions that could be considered in deliberating and resolving challenges. It is our hope that this article serves as an educational resource for students, new and experienced investigators, and Human Subjects Review Board members regarding some of the complexity of issues of fairness, equality, diversity, and personal rights for preventive science research and practice.

## Need for Ethical Guidelines in Prevention Science

Ethical guidelines for prevention research and practice are currently informed by a multiplicity of research, professional, and discipline-based codes of conduct, value statements, and standards (e.g., medicine, nursing, psychology, education, anthropology, criminology, public health, sociology, and social work). These guidelines have much to offer to prevention scientists engaged in implementation activities. However, to date, the links between ethical guidelines and prevention science activities have not been made explicit, and specific guidelines have not been articulated or deliberated within the field of prevention science.

Past discussion of ethics in community-based or participatory-action research has also identified and considered ethical issues related to the evaluation or implementation of evidence-based programs for vulnerable communities and populations (Cargill et al. 2016; Hiriscau et al. 2014; Tamariz et al. 2015) or children and youth (Leadbeater et al. 2006). Community psychology has also discussed the vast array of professional skills and methods related to real-world efforts to implement preventive interventions (e.g., Campbell 2016; Moritsugu et al. 2016; Robinson et al. 2017). Indeed, almost three decades ago, Trickett (1998) summarized the, then current, discussion of “ethical issues in the protection of communities involved in primary prevention projects” (p.321), noting that “the range and scope of such activities are far too varied, and the accumulated professional wisdom about ethical issues is too disjointed” (p. 323) to prescribe ethical codes. Trickett (1998) also set the stage for further discussion by locating ethical concerns at the intersection between science and practice by suggesting that

Community interventions involve much more than negotiating with stakeholders to implement a technology; rather, they involve a confrontation among often competing values, competing groups, and … competing cultures. Further, they are often based on scientific evidence that is limited; external funding that is finite and creates its own community dynamics; and the agendas of the interventionist, which include both community interests and self-interests. (p. 323)

Trickett’s comments come close to characterizing the current state of affairs for the field of prevention science and the need to identify and discuss ethical issues in the various phases of implementation of evidence-based programs (e.g., promotion and adoption of programs, adoption, fidelity monitoring, scale-up, and replication). The ethical challenges that can emerge from the relationships between prevention scientists and community stakeholders are often still dealt with “on the go” in the absence of specific ethical guidelines. Identification and articulation of the ethical issues that may be salient in the implementation of preventive interventions can serve multiple purposes, including (1) guidance for action as prevention researchers and practitioners and stakeholders encounter similar ethical challenges, (2) a framework for training students who are initiating careers in prevention science, and (3) examples and principles that stimulate discussions, which in turn, successively improve the beneficence and effectiveness of prevention practice. In the section that follows, we summarize the work and findings of the SPR Ethics Task Force, with the goal of informing efforts to articulate ethical practice and spur ongoing discussion of ethical issues for prevention scientists.

## SPR Task Force on Ethical Issues in Prevention Science

The goal of the SPR Task Force on Ethical Issues in Prevention Science was to identify prominent ethical issues that are encountered by prevention researchers and practitioners engaged particularly in dissemination and implementation activities. These include activities that may not be considered under the purview of existing ethical reviews of research by Institutional Review Boards or funding research institutes. From the onset, the Task Force agreed that the first step was to explore whether or not the Society of Prevention Research membership could identify ethical dilemmas that were not well covered by existing ethical guidelines (e.g., see American Psychological Association (APA, 2014) ethics code). The second step was to consider the need for such guidelines and standards, which necessarily entails a more inclusive deliberative process. To accomplish the mission of identification and articulation of potential ethical challenges, the Task Force took several steps.

Initially, the Task Force surveyed SPR members to elicit the experiences of prevention scientists and practitioners that posed ethical challenges. A roundtable discussion, led by Irwin Sandler, Tom Dishion, John Lochman, Ken Dodge, Brian Bumbarger, and Catherine Bradshaw, was conducted at the 2015 SPR Annual Conference. The roundtable discussion was well attended, and members drew attention to at least 50 specific situations that they believed involved ethical dilemmas. The Task Force members—the co-authors of this article—met in person and by phone several times in the winter of 2016. It was apparent early in the Task Force discussions that ethical issues involving efficacy trials in prevention science are included in extant professional and scientific ethical guidelines. However, ethical issues related to the activities that are necessary for the implementation and scale-up of EBPs were less well explicated. The Task Force worked to achieve a consensus to reduce the 50 situations identified in the roundtable discussion to distinct activities in which ethical dilemmas could emerge in the course of implementing EBPs (see Table 1). Seven activities were identified. At the 2016 SPR Annual Conference, an additional roundtable discussion elicited additional feedback from the SPR membership on the salience of the seven implementation activities. Participants rated the “importance” of the ethical challenges in these activities on a scale of 1 to 10. The ratings (range 6.9 to 8.7) recognized that each of the seven activities involve major opportunities for advancing public good or community well-being, in addition to potential ethical dilemmas.

**Table 1:** Examples of prevention science activities involved in the implementation of evidence-based interventions and related ethical challenges

Activity of prevention scientist or professional	Balancing potential benefits with ethical challenges	Relevant core ethical principles
1. Consulting with communities, institutions, and public agencies regarding selection of evidence-based preventive interventions for implementation	Potential for improving public welfare of individuals and communities, with the interest of the consultant	Beneficence, non-maleficence, autonomy, and conflict of interest (transparency)
2. Forming contractual or collaborative relationships with communities or public agencies to implement or scale-up EBPs	Potential for improving public welfare through improving the quality and effectiveness of services, with local control of change processes and equity in distribution of resources	Autonomy, social justice, integrity, conflict of interest
3. Implementation of EBPs that involve youth, disadvantaged groups, minorities, immigrants, and aboriginal peoples	Balancing interest of implementation access and fidelity, with need for accurate information to empower and promote best interests and respect for self-determination of vulnerable participants	Respect for persons and cultural difference, concern for welfare, social justice
4. Balancing implementation fidelity and adaptations with community needs and resources	Balancing desire for fidelity with adaptations that meet community needs for and access to evidence-based resources	Social justice, autonomy, transparency
5. Linking or accessing publically available data in the absence of consent	Balancing public welfare and social justice (access to benefits of research), ecological validity, and reduced participant burden, with respect for individuals’ confidentiality, autonomy, and self-determination when using multiple data sources in implementation evaluations	Confidentiality, respect for individual’s privacy and autonomy, transparency, conflict of interest
6. Building capacity to implement and scale-up EBPs through commercialization	Balancing remuneration to support intervention and implementation, with access and the interests of communities, institutions, and public agencies	Conflict of interests, social justice, beneficence
7. Facilitating replication by independent groups	Balancing interests of intervention developers, with benefits of replication research	Autonomy, fidelity, conflict of interests

## Seven Common Activities of Prevention Scientists Engaged in the Implementation of EBPs

We briefly elaborate and give examples of each of the seven activities that were identified through the SPR Task Force’s progressive process. In Table 1, column 1, we identify an activity of prevention researchers and practitioners. In column 2, we identify both the public good likely to result from the activity and ethical dilemmas that could arise. In column 3, we identify ethical principles from multiple fields that can be applied to guide ethical behavior.

Core ethical principles are interdependent and transcend specific disciplines. They are at the root of the collective understanding and ethical decision-making, and are uncontroversial in their generality. By definition, ethical challenges are dilemmas or hard choices that call into question how best to balance ethical principles and moral values in the context of specific actions. For example, the APA (http://www.apa.org/ethics/code/) provides definitions for the following five core aspirational principles that are at the foundation of ethical guidelines for psychologists: beneficence and non-maleficence, fidelity and responsibility, integrity, justice, and respect for peoples’ rights and dignity. Given the trans-disciplinary nature of prevention science, the principles emphasized in the ethical codes of several scientific and professional organizations are also informative (see Table 2).

**Table 2:** Disciplined-based professional ethics resources

American Psychological Association http://www.apa.org/ethics/code/	
Code of Ethics of the American Public Health Association http://www.phls.org/CMSuploads/Principles-of-the-Ethical-Practice-of-PH-Version-2.2-68496.pdf	
British Society of Criminology www.britsoccrim.org/docs/CodeofEthics.pdf	
Academy of Criminal Justice http://www.acjs.org/pubs/167_671_2922.cfm	
American Society of Criminology Code of Ethics http://www.asc41.com/code%20of%20ethics%20copies/ASC_Code_of_Ethics_Draft.pdf	
National Association for Social Work http://www.socialworksearch.com/html/nasw.shtml	

### Activity 1. Consulting With Communities, Institutions, and Public Agencies Regarding Selection of Evidence-Based Preventive Interventions for Implementation

Researchers who have a significant role in the development and evaluation of specific programs are frequently involved in consulting on the selection of EBPs for implementation and scale-up. Such dual roles are often complementary and can help to ensure implementation fidelity, as well as access to program resources, technical assistance, and evaluation tools. However, dual roles can also lead to conflicts of interest and ethical dilemmas when the scientist directly benefits from the implementation or scale-up (e.g., being compensated financially or in terms of enhanced status or reputation). Conflicts of interest can lead to overstating effects or to minimizing limitations or negative findings of existing evaluations of the researchers’ own program and understating the impact of alternative programs, or failing to identify other programs that may meet the needs of the communities, institutions, or agencies. Failure to clearly state conflicts of interest, in turn, undermines the autonomy of the stakeholders in decision-making. To make fully informed and free choices of best practices for their communities, stakeholders or representatives need accurate and complete information about available choices, adequacy of the evidence supporting a program, and initial and continued costs before adopting or scaling-up a specific preventive intervention.

#### Example

A prevention scientist is on an advisory board to a Governors’ council that has been charged with improving the quality of state services for children and families that support mental health and prevent substance use. The prevention scientist is also the CEO of a non-profit agency that disseminates and provides technical assistance on a specific family-centered program that she developed, evaluated, and trademarked. Based on her long-term relationship with the board members, her familiarity with the intervention, and prior evidence of its effectiveness, she would like to enter into a contractual arrangement to scale-up her program across the state. Despite the strong evidence in favor of her program, she is concerned that there is a potential conflict of interest, as she does not have the knowledge base to present in detail other potential EBPs.

In addition to the obvious ethical tension, the reality of the situation is that the relationship between the program developer and the community can present a powerful opportunity leading to community benefits. Questions for consideration here relate to the researcher’s prior relationship to the board, conflict of interest, the power or authority derived from perceived expertise, and respect for the autonomy (free and informed choice) of the community stakeholders. A number of questions are relevant to addressing this ethical dilemma. For example, what benefits will the consulting scientist obtain? What other programs address the same problems and what is the scientific evidence for their efficacy? Can another scientist present a review of available programs that can address the board’s needs? How do the costs, accessibility of resources, appropriateness, and likelihood of positive effects compare across programs? It is possible to reduce the ethical tension of this situation by declaring conflict of interest, directing community stakeholders to alternative information sources that can inform decisions leading to major investments, and disclosing potential pitfalls and benefits of an EBP.

### Activity 2. Forming Contractual or Collaborative Relationships With Communities or Public Agencies to Implement or Scale-Up EBPs

Prevention scientists in general and program developers in particular have invaluable expertise that can inform community stakeholders’ efforts to implement and scale-up EBPs. However, these relationships are potentially complex both in how they are defined initially and in how they change dynamically through the various stages of implementation of a chosen EBP.

Some collaborations are initiated by community request to solve a problem or address a need that they have identified. In other cases, scientists who are evaluating scale-up or implementation of an evidence-based program with a track record of efficacy initiate these relationships. In both conditions, balancing community needs with sound methods for evaluation can be challenging. This concern is particularly true, for example, for selective and indicated interventions in which communities, schools, or individuals are recruited because of their high-risk status. Often, study participants are recruited by appealing to their need to address a particular problem or risk and a specific program or practice is represented as likely to be effective for addressing their problem or risk. If the program requested or offered has already been demonstrated in rigorous research to produce positive benefits, as has been implied during the recruitment, the preferred research design involving randomizing participants into a treatment or no treatment control group may be unethical because it will result in a denial of the known benefits to one group. If the program requested or offered has *not* yet been demonstrated to be effective for reducing the problem, a recruitment strategy that implies that benefits will accrue from study participation appears unethical. The challenge is to tailor both the participant recruitment strategy and the research design to the situation. If little is known about the effects of the program with the targeted population, a randomized experiment with a no-treatment control group is the preferred design, but the subject recruitment strategy must accurately describe the state of current knowledge about the effects of the program. If positive effects have already been clearly demonstrated with the targeted population, a research design involving a no-treatment control group would appear unnecessary and potentially unethical. Alternative designs could be considered. These may involve, for example, assessing different levels of the natural course of implementation, assessing different implementation strategies (standard training compared to standard training plus ongoing coaching and feedback), or comparing implementations offering standard and community-adapted versions of the same intervention.

In fact, ethical challenges can be encountered at any step or phase of the often lengthy course of implementation of EBPs. These include early phases of consultation, relationship building, and program selection, as noted in the previous section, as well as later stages that focus on activities related to adapting, sustaining, and evaluating the impact of the intervention. Hence, it is not the case that all concerns are likely to be recognized and solved at the beginning of the process; rather, ethical challenges need to be anticipated and decisions that are made may need to be revisited as the implementation process unfolds. Again, the prevention scientist as both program developer and implementer may encounter conflicts of interest if personal benefits are accrued in the scale-up process. Conflicts can also arise when scientists are acting on behalf of both funders and communities. Several potential ethical issues were raised, in the course of the Task Force’s work, in relation to ongoing consultation for the implementation of a specific program, which we reduced to two examples.

#### Example 1

A prevention scientist is employed as a consultant by a state agency to assess the effectiveness of an intervention targeting mental illness and substance use in several communities. Findings are mixed, and some agencies implemented the intervention with considerable fidelity, whereas others did not. One agency that shows particularly poor implementation and results acknowledges the problems, asks the consultant not to report the findings in the final report or in publications because of concerns that the agency will lose future funding and support from the state. The consultant is challenged, as he does not wish to harm the agency with poor implementation outcomes, but endorses the conventional standards of integrity in reporting scientific results. The consultant is also concerned that the poor outcomes for one agency reflect poorly on the EBP he developed.

Discussion to articulate and anticipate partnership agreements at the outset of implementation partnerships may be vital to moving implementation efforts forward and avoiding costly impasses. Questions to consider include the following: How are the identity or confidentiality of specific communities protected? What are the limits of confidentiality? How will agreements be reached about the reporting of unexpected or negative findings? What lessons can be learned about implementation readiness or successes? Would other communities benefit from the knowledge gained by considering the success and failures of implementation efforts? What procedures can be established to address conflicts between scientists and participating communities if they do occur in the course of implementation?

#### Example 2

A partnership has been formed between a prevention scientist and a national non-profit agency to implement and scale-up an EBP. The agreement involves the prevention scientist's training agency staff to deliver the EBP to their clients. Funding is available to support the initial stages of the collaboration including consulting on EBP adaptations, evaluating the outcomes of implementation and training staff to fidelity. Results from the evaluation are positive, but the agency is struggling with staff turnover and leadership changes. The agency requests additional support from the prevention scientist to sustaining the EBP and implementation fidelity, but it does not have adequate funds to support these consultations. The prevention scientist has mixed feelings about continued involvement. She sees the potential value of her continued involvement with the community to sustain program uptake and fidelity, on the one hand, but on the other hand, she is unable to continue in these efforts without financial support for her continued work.

Questions for consideration in this scenario relate to the continued involvement of the scientist after an implementation trial. What are the best practices for implementation consultants with respect to enabling agencies to acquire the support they need to sustain positive change, without incurring unrealistic costs? What is the obligation of the implementation team to support continued efforts of the non-profit to train its staff and deliver the intervention? Could best practices involve proactive discussion and planning for sustainment of the program following the availability of support for the program developer? What is the implied obligation of the prevention scientist(s) to assist in the sustainment of the program? What is the ongoing obligation in the absence of financial remuneration? Should the implementation team expect payment for their continued involvement? What are the best practices for implementation consultants with respect to enabling agencies to acquire the support they need to sustain positive change, without incurring unrealistic costs?

### Activity 3. Implementation of EBPs That Involve Youth, Disadvantaged Groups, Minorities, Immigrants, and Aboriginal Peoples

A core mission of prevention science is to apply the knowledge gained to the benefit of vulnerable groups, to promote health and well-being, and to reduce disparities. Historically, there are many examples in which disadvantaged groups have been either excluded from research (and thus from its potential benefits) or in which they have been included but have neither been adequately informed about risks to them nor compensated for their participation. Implementation of EBPs in partnerships between prevention scientists and organizations that speak to or provide services for disenfranchised or vulnerable populations (e.g., groups who are vulnerable due to age, poverty, disabilities sexual orientation, or minority, immigrant, or aboriginal status) pose particular challenges. When prevention scientists consult with agencies that serve vulnerable populations, there is a heightened need for anticipating and articulating concerns (e.g., data ownership, reporting requirements) and benefits, and for adhering to ethical practices when forming collaborations with disadvantaged groups.

#### Example 3

A program developer is working closely within an aboriginal community advisory board to achieve funding to implement a family-centered intervention that has been extensively evaluated with non-aboriginal populations. After receiving funding, the advisory board revises and adapts much of the original program without directly consulting the program developer. Some of the core features of the program were removed from the original EBP because of incongruence with the culture and values of the aboriginal community. After 2 years, the community reports high engagement rates using the revised program. The community advisory group again seeks support from the program developer in order to apply for ongoing funding. The community advisors are reluctant to agree to an evaluation of the adapted program because they believe that this would attenuate the trusting relationship between families and the health agency serving the community. They fear that collecting data could violate privacy for needy families and reduce the reach of the program threshold. The program developer is concerned about the lack of evidence of effectiveness for the adapted program and the communities’ reliance on past evidence related to the original program to support their claims of effectiveness. He is conflicted about the most ethical course of action in reapplying for funding.

There is a pressing need for greater expertise, in general, on how EBPs can best be adapted to fit some communities. The example reveals several issues. Who are the representatives of the group targeted (parents, elders, policy decision-makers)? Who speaks for the members of vulnerable communities? How can openness, transparency, reliability, accountability, and reciprocity be assured between prevention scientists and members of vulnerable communities? Can the positive or negative effects of the evaluation outcomes be anticipated? Whose responsibility is it to develop implementation and evaluation procedures that do not conflict with core values of some cultural communities? What mechanism can be put in place to manage any conflicts in ongoing partnerships?

### Activity 4. Balancing Implementation Fidelity and Adaptations With Community Needs and Resources

In the course of scientist-driven and funded implementation efforts, resources are often available to assess and monitor implementation to ensure that providers deliver interventions with adequate adherence and competence. Yet, many factors can affect fidelity in the implementation of interventions. Fidelity, itself, has many components (e.g., adherence, quality, dosage, participant engagement, differentiation from similar intervention, adaptations) that can each affect the outcomes of the intervention (Berkel et al. 2011; Hansen 2014). In the implementation and scale-up of interventions, prevention scientists may be particularly aware of the difficulties in maintaining fidelity of the intervention to achieve desired outcomes. The effectiveness of evidence-based interventions typically depends on the implementation quality and capacity of core program components (that are often unspecified or unknown). Fidelity and user adaptations were once seen as opposite ends of effective implementation. However, acknowledging the limits of generalizability of a program developed in one community for use in another is also important (Chambers et al. 2013). Some adaptations, particularly those that do not detract from the delivery of core program elements, can enhance user buy-in and local or cultural relevance of the intervention (Van der Kreeft et al. 2014; Zayas et al. 2012). However, communities also may incur opportunity costs when adaptations that are made due to cultural differences or lack of resources result in poor intervention effects. More prevention research is needed to examine the effect of cultural adaptation while controlling for other differences in duration or intensity of the intervention.

Ethical issues can arise in the tensions between the need for fidelity in implementation quality and real-world practice. For example, communities may not have the resources to scale-up an intervention with fidelity, and the impact of the community’s efforts may be negligible at best, or iatrogenic at worse, because of low implementation quality. Adaptations may be motivated by the implementer’s desire to more closely align the intervention with the consumers’ needs and preferences or to increase feasibility and thus likelihood of its sustainability. However, local adaptations are rarely empirically informed, they are almost never documented or assessed, and they may be made without consultation with the developers. This raises questions about the obligations that prevention scientists may have to train implementers in the core features of a program and their latitude in adapting core features. Does the scientist have a responsibility to monitor local adaptations to ensure that the intervention consumers receive includes the intervention’s core components? What are the limits of the scientists’ responsibility?

#### Example 4

A prevention scientist is the director of a non-profit agency that is the purveyor of a specific family-centered intervention. The director of a community-based organization that is located within an inner-city, poor community approaches the prevention scientist to contract with his agency to implement an intervention. The director explains that this particular intervention fits the needs and preferences of the community very well and the providers employed at the organization are highly skilled and motivated. However, they plan to implement a shortened version of the program to enhance the number of families who can receive the program. The community organization resources are very limited, so they cannot afford to monitor implementation or collect any implementation-related data. To support the non-profit agency’s positive intentions to promote the EBP, the prevention scientist considers consulting with the agency despite the lack of evidence for the shortened program, and the lack of fidelity monitoring.

The example raises concerns related to ethical principles of respect for autonomy of the stakeholders, accessibility of the program by disenfranchised populations, and beneficence versus the prevention of harm. The tension between implementation rigor, adaptations, and data collection capacity of community-based organizations introduces several questions with ethical implications: What is the evidence for the core features of the EBP? Does the prevention scientist, who is also a program developer and purveyor of intervention, have a responsibility to ensure that community organizations monitor the implementation to ensure that it is delivered as intended to enhance benefits and reduce negative or iatrogenic effects? Does the scientist have a responsibility to include feasible, efficient methods to help the organization monitor or evaluate an implementation before making programs available for dissemination? If the prevention scientist forms a contract with the community organization to deliver the intervention, does the prevention scientist or community organization have an obligation to inform the consumers that they may or may not receive the active intervention components? Should the prevention scientist only make the intervention available if the community organization agrees to implement on a smaller scale and reallocate resources for a more robust assessment of implementation, even if this approach means that fewer families will have an opportunity to participate?

### Activity 5. Linking or Accessing Publicly Available Data in the Absence of Consent

Increasingly, administrative (e.g., medical, hospital, or court, education records, vital statistics) data are being used in prevention science, particularly in the evaluation of monitoring changes in public welfare that could be attributed to the scale-up of an EBP. Administrative data are typically collected as part of government documentation and service delivery in sectors such as education, health, welfare, justice, and labor. The quality of administrative data has historically been poor, but it has improved with advances in technology and demands for use. Examples include birth records, death records, education records, child protective service files, juvenile justice files, divorce records, hospital data, medical claims, and tax records. Such data can be a valuable source of information about individuals and their contexts. The data can also offer unparalleled advantages for including entire populations, often with little missing data, high accuracy, low bias and costs, and low participant burden. Policies such as the *Health Insurance Privacy and Portability Act* (*HIPPA*) and the *Family Educational Rights and Privacy Act* (*FERPA*) protect individuals to some extent, but they may not address the use of administrative data for implementation research despite the potential public benefits. Often, it is required to establish a HIPAA Waiver of Consent and an HIPAA Waiver of Authorization to gain access to the required data for surveillance or evaluation purposes.

Secondary analyses of existing administrative or existing research data can also be used to assess the outcomes of preventive interventions or to corroborate or critique published findings. Inclusion of administrative data can also extend the questions that can be answered from existing longitudinal data and evaluations. For example, administrative data can inform prevention scientists about long-term outcomes of interventions and extend the value of longitudinal data (e.g., linking high school performance data to distinguish youth most likely to benefits from post-secondary promotion programs). The use of administrative data can also limit the burdens of research for participants, be highly cost-effective, and improve generalizability to large representative populations.

When the public health benefits are clear, ethical concerns related to secondary analyses of de-identified or limited data sets (obtained and protected) through agency established processes are often minimal. However, some benefits are accrued only when secondary analyses are linked to individuals’ identifiers (for example, when public data are linked to existing longitudinal data sets). When consent was given for the original purposes for collecting the data, but not for the use of the linked data, considerations of the need to balance potential benefits of the research with respect for individuals’ autonomy and self-determination are of concern. Privacy laws and data security standards that govern the use of existing data also need careful consideration. For some data, gaining individual’s consent may be possible. However, obtaining active consent can also be impractical or even harmful, for example, if the population is widely dispersed or deceased, when resources and manpower to contact the individuals are costly, or confidentiality or identity as a research participant would be compromised in the process of re-consenting individuals*.*

#### Example 5

A prevention scientist who randomized children in a community and implemented an early childhood prevention program found predictable short-term benefits to the children and families. Twelve years later, the prevention scientist would like to see if the short-term benefits extend to long-term outcomes that are meaningful to the participants and the community that promoted the prevention study. Public data are available for the individual participants in the intervention and control groups (e.g., high school grades, achievement scores, and graduation dates). Recently, government employees agreed to link the earlier consented data with publicly available high school graduation records on file at the state level. Consent was provided for families to participate in the trial and to provide data for the target child up to age of 3, but accessing publicly available data was not mentioned in the consent forms.

Several questions for discussion can be considered to illuminate potential conflicts between the benefits of research and the privacy of individuals in relation to the needed administrative data linkages. Policies and procedures on linking publicly available data with consented data may vary across universities, state-level government agencies, and national settings. While most existing guidelines are silent on this issue, the Canadian Tri-Council Policy Statement on Ethical Conduct for Research Involving Humans (2014, p. 64) explicitly suggests considerations that are relevant to the using administrative data. Is identifiable information essential to the research? What are the benefits of information to public health and well-being or to understanding the benefits or effects of the proposed linkages? Is the use of identifiable information without the participants’ consent unlikely to adversely affect the welfare of individuals to whom the information relates? How will the planned use of the data ensure individuals’ anonymity? What measures protect the privacy of individuals, and safeguard the identifiable information? Can institutions that safeguard administrative data provide de-identified data linked to existing data sets? Can the researchers comply with any known preferences previously expressed by individuals about any use of their information? Did the individual opt out of the use of their data for research purposes when it was given? Is it impossible or impractical to seek consent from individuals to whom the information relates (for example, if they had moved or died)? Would identifying the individual as a member of the data set in the course of re-consenting create harm or violate the individuals’ confidentiality? Who owns the linked data file and is responsible for maintaining data integrity and compliance with privacy laws? Who is responsible for establishing, monitoring, implementing, and revising data user agreements between all parties involved? Who has access to the linked data files versus limited access to aggregate data? Have the researchers obtained other necessary permissions for secondary use of information for research purposes?

### Activity 6. Building Capacity to Implement and Scale-Up EBPs Through Commercialization

A key strategy for building the capacity to support dissemination and implementation of EBPs is to trademark and commercialize the practice for public use. A clear advantage of commercialization is it can provide revenue from use of the intervention, training, assessment, and support services for growing the capacity to scale-up the intervention. Although research funding often supports program development and evaluation, it is less often available for the dissemination of public health or preventive interventions. Thus, commercialization can also increase funding needed to market the resource to users, make training available, and enhance the capacity to monitoring of fidelity and outcomes.

At the same time, commercialization can create conflicts of interest for prevention scientists working with communities and organizations (Caulfield & Ogbogu 2015). When charges for intervention protocols, assessments, and support services exceed the budget of schools or community agencies, access to programs by disadvantaged groups may be restricted. Restrictions of use due to intellectual property and copyright issues may further limit access and use of EBPs in community settings with low resources. Commercialization of an intervention can create marketing pressures that can be seen in claims that overstate the scope of findings of an intervention or incomplete disclosures of effects as compared with competing programs (Caulfield & Ogbogu 2015). Premature commercialization can erode public confidence if expectations are not realized. Replication research can also be limited when commercialization makes an intervention a proprietary product that others cannot access without cost and the consent of the owner. Scientific study of the preventive intervention can be restricted if the owner does not allow others to independently replicate the effects or to study program effects in comparative effectiveness trials.

#### Example 6

A community agency hired a prevention practitioner with extensive training and certification in a commercially available evidence-based preventive intervention for parents of children with behavior problems. Over five years, the practitioner trained most of the agency supervisors to implement the intervention. Parent reports indicate some success in reducing child behavior problems. The agency is interested in expanding its services, but due to their financial constraints, it would be cost-effective for the agency to expand the supervisors’ role in training new providers and monitoring fidelity, rather than paying the cost of the program-certified practitioner. The prevention practitioner is consulted about the plan and advises the agency that they may be infringing on the copyright of the program. She is conflicted about whether or not the program developer should be informed of the potential copyright violation and wonders if the program continues to meet the developers’ certification standards.

Several questions are raised about conflict of interest of the developer, autonomy of the users, and social justice or accessibility of the intervention across socio-economic groups. What are the limits of the users’ obligation to pay for the most effective program? The user agency is on a very limited budget. Can the agency continue to use the program independently and train new providers or do these actions infringe on copyright if the agency does not renew their site license annually as is required by the commercialized program? What are reasonable costs for renewing the license? How can the commercial value of an intervention be assessed if it is directed at users who have difficulty paying? If the developer used public funding to create and evaluate the program, is there an obligation to provide free access to the resources? What are the consequences of open access for promoting implementation fidelity? What role can the prevention scientist play in helping the agency to consider its options?

### Activity 7. Facilitating Replication by Independent Research Groups

Due to the complexities of procedures, training, and assessment protocols, replication studies of EBPs with new providers or in diverse communities often involve the program developer in a variety of roles. Developers can facilitate the rigor of the replication study by mapping precise research procedures, providing access to program resources, training to ensure implementation fidelity, and data sharing. Independent replication studies conducted by new research teams that do not involve the program developer can also add important information about the conditions under which an EBP will be effective, or about what is needed to implement the program with fidelity in real-world settings, or what can enhance the generalizability of the outcome effects shown in developer-driven evaluations (see Gottfredson et al. 2015). Replication studies can also provide estimates of developer tendencies to emphasize positive effects to the neglect of null effects, or potential iatrogenic effects under some conditions. On the other hand, when EBPs are not well understood, training is inadequate, implementation is poor, or the study lacks adequate controls, the outcomes of the replication can be confusing to the field and potentially set back progress in dissemination of EBPs.

#### Example 7

The developer of a school-based EBP is asked to provide the training manuals, fidelity measurements, and procedures for an independent research team to conduct a replication study. The research team is interested in an independent evaluation and is not seeking assistance from the developer. The research team is well trained in prevention and implementation science, and has published prevention research involving school-based prevention strategies. The program developer values independent replication but is concerned that the research team will not implement the EBP with fidelity, without extensive support and consultation. He doubts the schools’ readiness to implement the program and is concerned about possible needed program adaptations for the population to be recruited. He fears that the reputation of the EBP will be harmed by the lack of effects resulting from poor implementation, and wonders if it would be best for the reputation of the EBP if he withdraws support for the replication effort.

The conditions that entail a solid replication involve harmony between an array of procedures and ecological conditions. The investment of a single prevention study often involves years of a prevention scientist’s career and considerable efforts to maintain funding for development and evaluation. Thus, caution is natural in consenting to independent replications that may involve investigators who are less invested in the details of the prevention protocol, or who have less control over how it is implemented. Ethical questions also arise concerning who controls the intellectual property that emerges from prevention science research. How do we promote replication studies if core EBPs are unavailable or too costly to use? What conditions on the collaboration for replication are justified? What costs could be incurred and who will pay for them? Is the program adequately developed to support implementation fidelity in the absence of the developer? Can the practitioner groups’ readiness to implement the intervention be assessed? Are tools for fidelity monitoring available and clear? Will the engagement of populations not previously involved and adaptations be documented?

## Summary: Gaps in Understanding and Navigating Ethics in Implementation Activities

Ethical dilemmas, by definition, involve difficult decisions and may not have ready or uncontroversial solutions, and ethical guidelines for scale-up and implementation are particularly lacking. The preceding description of the diversity of implementation activities reveals many gaps in our understanding of how to navigate potential ethical challenges that warrant further discussion. Traditionally, ethical guidelines are written to balance the benefits of research with respect for the autonomy and rights of *individuals* participating in research rather than for promoting public welfare or well-being, with the notable exception of guidelines for public health practitioners (see Thomas et al. 2002). As noted in the activities discussed, implementation activities are linked to typically long-term, trusting relationships with community stakeholders who represent individuals within the community who are the targets of the intervention. Thus, implementation of preventive interventions shifts ethical concerns from the need to balance the potential benefits of an intervention with respect for the autonomy, rights, and benefits of individuals to the need to consider individual rights in the context of community action and public welfare (Bromley et al. 2015; Goodyear-Smith et al. 2015). The current article is written from the perspective of prevention scientists who work with communities. Although there were community stakeholders at the SPR roundtable discussion, the majority of the participants were prevention researchers. We anticipate that directly involving community stakeholders in both the generation of ethical dilemmas, as well as articulating costs and benefits of specific courses of action, will enrich the discussion and promote healthy partnerships between prevention science and implementing communities.

Further complicating the implementation of EBPs, the scientific evidence for specific EBPs comes primarily from specific efficacy trials with limited populations. Activities or processes for generalizing or adapting EBPs to real-world scale-up activities with diverse populations are complex and can create ethical challenges. In efficacy trials, community conditions, individual participants, and intended outcomes are, to the extent possible, defined in advance. The effects obtained as programs go to scale in community settings may not match those found in investigator highly controlled efficacy trials (Weisz et al. 2014). In the course of implementation and scale-up activities, adaptation is the rule rather than the exception (see Chambers et al. 2013). A program developers’ involvement can be an asset to the successful application of an EBP to the specific needs of a community or agency, but the developers’ involvement can also create conflicts of interest for the developer. EBPs also often target widespread or “wicked problems” (Lavery 2016, p. 1; e.g., obesity, bullying, addictions, delinquency, violence, or health disparities and service inequities) that lack specific solutions offered by a particular intervention.

## Recommendations and Next Steps

In this article, we identify ethical challenges related to implementation activities of prevention scientists and practitioners and questions that could inform efforts to navigate challenges to maximize benefits and minimize harms. The work of the SPR Task Force identified the desire for further explication of the ethical challenges encountered in the course of implementation and scale-up of preventive interventions and of steps taken by experienced teams to identify, address and, where necessary, deliberate concerns. New investigators, students, and community representatives will benefit from knowing more about the specific experiences of seasoned scientists who have dealt with ethical challenges in the course of their implementation and scale-up activities. The exchange and discussion of experiences could continue to be supported in conference sessions. It may also be helpful to encourage commentaries on ethical challenge and their solutions in procedure sections of publications. Publication of articles or case studies of ethical challenges and actions undertaken during the implementation of EBPs and commentaries from the perspective of both scientists and program users about ethical challenges are needed. Sharing and documenting experiences will help to establish clear guidelines for ethical practice as the field of prevention science continues to evolve and mature. Initiating discussion and normalizing consultation with peers and colleagues around issues is also important.

In conclusion, we pose value statements summarizing learnings to date through the Task Force processes. These statements are listed below not as a prescribed list of guidelines but in order to make concrete steps forward that (a) encourage rejoinders and other articles to further flesh out the issues raised here, (b) foster discussion and debates among SPR members, and (c) promote efforts to decide whether standards or guidelines are warranted or possible at the current juncture in the development of prevention science. Although further deliberation is clearly needed, the discussion to date suggests some first steps towards making the values underlying ethical actions in the implementation of EBPs more explicit. Our work to date suggests that prevention scientists should

1. Be guided fundamentally by intent to maximize benefits and prevent harms to both individuals and public well-being and welfare.
2. Respect the rights of those whose lives they hope to improve and empower them to make decisions concerning issues that affect them.
3. Maintain high standards of transparency in representing themselves to stakeholders and in disseminating scientific findings related to evidence-based practices.
4. Provide accurate and complete information about the generalizability of available evidence, available choices, and costs of EBPs to enhance the capacity of communities to make informed decisions regarding their adoption or scale-up of preventive intervention.
5. Disclose financial and professional conflict of interests or limitations of expertise when presenting the scientific findings to stakeholders that affect program adoption, dissemination, and implementation strategies.
6. Promote *ongoing* communication, transparency, accountability, reliability, and reciprocity in relationships with all partners across the phases of implementation of preventive interventions.
7. Anticipate and respect diverse values, beliefs, and cultures of the community or population engaged in implementing an intervention.
8. Recognize that power is derived from informal and formal authority in relation to stakeholders and respect the autonomy of communities and their members.
9. Make data and complete results (significant and not significant) from prevention trials or implementation studies available to other scientists and stakeholders.
10. Recognize a responsibility to the community of prevention scientists to maintain positive relationships with their partner communities, institutions, and public agencies in order to support future partnerships and the continued practice of seeking out research-informed solutions to improve population well-being.

In closing, we return to Trickett’s (1998) concern about prematurely articling ethical standards for a relatively new field of study or practice. Discussion within the task force as well as feedback from reviewers of earlier drafts of the work of the Task Force indicated the need for further input to advance the next step of prescribing ethical guidelines or standards. Ongoing discussion is also needed to inform what levels of guidance the field of prevention science needs and wants to adopt (i.e., principles, standards, or guidelines) to support the ethical actions of prevention scientist and practitioners in their expanded research and non-research roles in implementing EBPs.

## References

[CR1] American Psychological Association (2014). Guidelines for prevention. American Psychologist..

[CR2] Berkel C, Mauricio AM, Schoenfelder E, Sandler IN (2011). Putting the pieces together: An integrated model of program implementation. Prevention Science.

[CR3] Bromley E, Mikesell L, Jones F, Khodyakov D (2015). From subject to participant: Ethics and the evolving role of community in health research. American Journal of Public Health.

[CR4] Campbell R (2016). It's the way that you do it': Developing an ethical framework for community psychology research and action. American Journal of Community Psychology.

[CR5] Canadian Institutes of Health Research, **N**atural Science and Engineering Research Council of Canada, and Social Sciences and Humanities research Council of Canada, Tri-council Policy Statement: Ethical Conduct for Research Involving Humans (2014) available on line at http://www.pre.ethics.gc.ca/eng/policy-politique/initiatives/tcps2-eptc2/chapter5-chapitre5/#toc05-1d.

[CR6] Cargill SS, DeBruin D, Eder M, Heitman E, Kaberry JM, McCormick JB (2016). Community-engaged research ethics review: Exploring flexibility in Federal Regulations. IRB: Ethics and Human Research.

[CR7] Caulfield T, Ogbogu U (2015). The commercialization of university-based research: Balancing risks and benefits. BMC Medical Ethics.

[CR8] Chambers DA, Glasgow RE, Stange KC (2013). The dynamic sustainability framework: Addressing the paradox of sustainment amid ongoing change. Implementation Science: IS.

[CR9] Clayton EW (2003). Ethical, legal, and social implications of genomic medicine. The New England Journal of Medicine.

[CR10] Conduct Problems Research Group (2010). Fast track intervention effects on youth arrests and delinquency. Journal of Experimental Criminology.

[CR11] Conway M (2014). Ethical issues in using twitter for public health surveillance and research: Developing a taxonomy of ethical concepts from the research literature. Journal of Medical Internet Research.

[CR12] Dymnicki AB (2014). Moderating effects of school climate on outcomes for the multisite violence prevention project universal program. Journal of Research on Adolescence.

[CR13] Fisher CB, Harrington McCarthy EL (2013). Ethics in prevention science involving genetic testing. Prevention Science.

[CR14] Goodyear-Smith F, Jackson C, Greenhalgh T (2015). Co-design and implementation research: Challenges and solutions for ethics committees. BMC Medical Ethics.

[CR15] Gottfredson DC, Cook TD, Gardner FM, Gorman-Smith D, Howe GW, Sandler IN, Zafft KM (2015). Standards of evidence for efficacy, effectiveness, and scale-up research in prevention science: Next generation. Prevention Science.

[CR16] Hansen W, Siboda Z, Petras H (2014). Measuring Fidelity. Defining prevention science.

[CR17] Hawkins JD, Jenson JM, Catalano R, Fraser MW, Botvin GJ, Shapiro V, Brown CH, Beardslee W, Brent D, Leslie LK, Rotheram-Borus MJ, Shea P, Shih A, Anthony E, Haggerty KP, Bender K, Gorman-Smith D, Casey E, Stone S (2015). Unleashing the power of prevention. Discussion Paper.

[CR18] Hiriscau IE, Stingelin-Giles N, Stadler C, Schmeck K, Reiter-Theil S (2014). A right to confidentiality or a duty to disclose? Ethical guidance for conducting prevention research with children and adolescents. European Child & Adolescent Psychiatry.

[CR19] Lavery JV (2016). ‘Wicked problems’, community engagement and the need for an implementation science for research ethics. Journal of Medical Ethics. August.

[CR20] Leadbeater BJ, Banister E, Benoit C, Jansson M, Marshall A, Riecken T (2006). Ethical issues in community-based research with children and youth.

[CR21] McCarthy P. (2016). Evidence at the Crossroads Pt. 7: Using Evidence To Do the Most Good, Even When it Reveals an “Inconvenient Truth” William T. Grant Foundatoin. Available at http://blog.wtgrantfoundation.org/post/136757855347/evidence-at-the-crossroads-pt-7-using-evidence.

[CR22] Mikal J, Hurst S, Conway M (2016). Ethical issues in using twitter for population-level depression monitoring: A qualitative study. BMC Medical Ethics.

[CR23] Moritsugu J, Vera E, Wong F, Duffy KG (2016). Community Psychology.

[CR24] National Research Council and Institute of Medicine. 2009. Preventing Mental, Emotional, and Behavioral Disorders Among Young People: Progress and Possibilities. Washington, DC: The National Academies Press. 10.17226/12480. 20662125

[CR25] Pisani AR, Wyman PA, Mohr DC, Perrino T, Gallo C, Villamar J, Brown CH (2016). Human subjects protection and technology in prevention science: Selected opportunities and challenges. Prevention Science.

[CR26] Price RH, Cowen EL, Lorion RP, Ramos-McKay J (1989). The search for effective prevention programs: What we learned along the way. American Journal of Orthopsychiatry.

[CR27] Robinson WL, Brown M, Beasley CR, Jason LA, Bond MA, Serrano-García I, Keys CB, Shinn M, Bond MA, Serrano-García I (2017). Advancing prevention intervention from theory to application: Challenges and contributions of community psychology. APA handbook of community psychology: Methods for community research and action for diverse groups and issues.

[CR28] Sandler IN, Wolchik SA, Cruden G, Mahrer NE, Ahn S, Brown H (2014). Meta-analytic review of preventive interventions for internalizing and externalizing outcomes. Annual Review of Clinical Psychology.

[CR29] Sloboda Z, Dusenbury, Petras H, Sloboda Z, Petras H (2014). Implementation science and the effective delivery of evidence-based programs. Defining Prevention Science.

[CR30] Tamariz L, Medina H, Taylor J, Carrasquillo O, Kobetz E, Palacio A (2015). Are research ethics committees prepared for community-based participatory research?. Journal of Empirical Research on Human Research Ethics.

[CR31] Thomas JC, Sage M, Dillenberg J, Guillory VJ (2002). A code of ethics for public health. American Journal of Public Health.

[CR32] Trickett EJ (1998). Toward a framework for defining and resolving ethical issues in the protection of communities involved in primary prevention projects. Ethics & Behavior.

[CR33] Van der Kreeft P, Jongbloet J, Van Havere T, Sloboda Z, Petras H (2014). Factors affecting implementation: Cultural adaptation and training. Defining Prevention Science.

[CR34] Weisz JR, Ng MY, Bearman SK (2014). Odd couple? Re-envisioning the relation between science and practice in the dissemination-implementation era. Clinical Psychological Science.

[CR35] Zayas L, Bellamy JL, Proctor EK, Brownson RC, Colditz GA, Proctor EA (2012). Considering the multiple service contexts in cultural adaptations of evidence-based practice. Dissemination and implementation research in health: Translating science to practice.

